# A narrative account of the key drivers in the development of the Learning from Deaths policy

**DOI:** 10.1177/13558196211010850

**Published:** 2021-04-25

**Authors:** Mirza Lalani, Helen Hogan

**Affiliations:** 1 Assistant Professor, Department of Health Services Research and Policy, London School of Hygiene and Tropical Medicine, UK; 2 Associate Professor, Department of Health Services Research and Policy, London School of Hygiene and Tropical Medicine, UK

**Keywords:** learning from deaths, multiple streams approach, policy entrepreneurs

## Abstract

**Objective:**

In recent years there has been a proliferation of patient safety policies in the United Kingdom triggered by well publicized failures in health care. The Learning from Deaths (LfD) policy was implemented in response to failures at Southern Health National Health Service (NHS) Foundation Trust. This study aims to develop a narrative to enable the understanding of the key drivers involved in its evolution and implications for future national patient safety policy development.

**Methods:**

A qualitative study was undertaken using documentary analysis and semi-structured interviews (n = 12) with policymakers from organizations involved in the design, implementation and assurance of LfD at a system level. Kingdon’s Multiple Streams Approach was used to frame the policymaking process.

**Results:**

The publication of the Southern Health independent review and subsequent highlighting by the Care Quality Commission of a fragmented approach to learning from deaths across the NHS opened a ‘policy window.’ Under the influence of the families affected by patient safety failures and the then Secretary of State, acting as ‘policy entrepreneurs,’ recently developed methods for mortality review were combined with mechanisms to enhance transparency and governance. This rapidly created a framework designed to ensure NHS organizations identified remedial safety problems and could be accountable for addressing them.

**Conclusions:**

The development of LfD exhibits several common features with other patient safety policies in the NHS. It was triggered by a crisis and the need for a prompt political response and attempts to address a range of concerns related to safety. In common with other safety policies, LfD contains inherent tensions related to its primary purpose, which may hinder its impact. In the absence of formal evaluations of these policies, deeper understanding of the policymaking process offers the possibility of identifying potential barriers to goal achievement.

## Introduction

In the United Kingdom (UK), over the last 20 years, there has been a proliferation of patient safety policy initiatives, performance measurement approaches and campaigns emanating from government, regulators and professional bodies. The history of patient safety in the UK clearly shows that against a backdrop of incremental efforts to improve the surveillance and management of safety across the National Health Service (NHS), it is often the impact of high profile health care scandals and the initiatives in response that lead to changes in policy direction.^1^

The Bristol inquiry into deaths of children with cardiac problems identified a hierarchical culture with limited attempts to learn from failures.^2^ The proposed solution was greater transparency across the NHS through the publication of comparable mortality statistics. The Mid Staffordshire inquiry revealed a reluctance from the organization to heed concerns raised by families and a lack of transparency when issues arose. This led to the Duty of Candour (DoC), a mandatory requirement for NHS staff to be open with families when failings in care arise.^3^

It is therefore unsurprising that development of national policy often diverges from a linear, rational, evidence-based process to follow a multifaceted and expedient approach, responsive to political and public demand for action. Gaining understanding of how policy is formed in relation to the specific circumstances and competing influences present during the decision-making process enables prediction of both positive impacts and potential negative consequences.

In March 2017, the NHS National Quality Board (NQB) published the *Learning from Deaths (LfD)* Framework. This followed a review by the national regulator for health and social care services (Care Quality Commission (CQC)), which highlighted poor systems across the NHS for organizational learning from deaths.^4,5^ Acute, community and mental health NHS Trusts were required to have their own LfD policy, based on the guidance, in place by September 2017. Organizations were expected to employ a systematic approach to reviewing deaths, to develop mortality governance and to engage with families of the bereaved (Table 1). In this study, we will develop a narrative account of the LfD framework using Kingdon’s Multiple Streams Approach (MSA),^8^ to enable us, through examination of its formulation, to consider the importance of patient safety focused policy agendas, changing priorities over time, prompts to action and consequences for potential impacts.

**Table 1.** An overview of the Learning from Deaths policy.

Learning from Deaths framework
The policy comprises three key components: mortality governance, a methodology for reviewing deaths and engaging with families of the bereaved.
Mortality governance: The policy requires Trust boards to adopt a series of principles and practices: reviewing deaths, reporting internally and externally (publication through quarterly Quality Accounts), sharing and acting upon learning from investigations, engaging with families of the bereaved and building the capacity of staff to undertake reviews. Boards are required to create a multi-disciplinary Mortality Surveillance Group which will facilitate these practices. Furthermore, the Trust board’s Non-Executive Director scrutinizes the mechanisms for reporting of mortality data and assures the publishing and sharing of relevant information about the organization’s achievements and challenges.
Methodology for reviewing deaths: The policy recommends the adoption of systematic case sampling and review of patient deaths using Structured Judgement Review (a robust and evidence-based methodology promoted by the Royal College of Physicians to make judgements on safety and quality of care within the National Mortality Case Record Review Programme) or other suitable tools.^6^ In instances in which deaths meet the threshold for further investigation, organizations will use the Serious Incident Framework^7^ to assist them in making such decisions.
Engaging with families: Bereaved families should be given several opportunities to raise concerns about care. Additionally, family involvement in the investigation of lapses of care should be sought, including reviewing reports. Furthermore, the policy emphasizes the importance of disclosure to families through the Duty of Candour.

## Methods

### Conceptual framework

The MSA conceptualizes policy agenda setting as comprising three streams. The ‘problem stream’ represents perceptions of public problems that are seen as requiring government intervention to address them. The ‘policy stream’ considers proposals for change, the potential solutions that emerge before problems reach the decision-making agenda. The ‘political stream’ comprises factors that shape rhetoric around problems and solutions, such as swings in national mood or campaigning from interest groups. These three streams may proceed independently of one another until at a specific point at which a ‘policy window’ may open, enabling a convergence. Window openings may be created by major events but may also be capitalized upon by ‘policy entrepreneurs’, who influence policy outcomes, sometimes for their own agenda.

### Study design

This study comprised two main components: analysis of relevant documents (Table 2) and semi-structured interviews with policymakers and other relevant stakeholders (n = 12). We selected reports from 2001 (publication of the Bristol inquiry)^2^ to March 2017 (publication of LfD), a period marked by growing public and political awareness of threats to health care quality. We focused on documents that identified drivers of patient safety policy especially in relation to mortality. The documentary analysis was informed by the relevant academic literature associated with mortality and safety in health care organizations.^9–13^

**Table 2.** Inquiry reports, reviews and responses, listed by date of publication.

Document title	Publication date	Document type	Author
*Learning from Bristol: The report of the public enquiry into children's heart surgery at the Bristol Royal Infirmary 1984–1985*	July 2001	Investigation report	Kennedy, I.
*Learning from Bristol: The Department of Health's response to the report of the Public Inquiry into children's heart surgery at the Bristol Royal Infirmary 1984–1995*	January 2002	Government response	Department of Health
*Report of the Mid Staffordshire NHS Foundation Trust public inquiry*	February 2013	Investigation report	Francis, R.
*Patients first and foremost – initial government response to Mid Staffordshire NHS Foundation Trust public inquiry*	March 2013	Government response	Department of Health
*Review into the quality of care and treatment provided by 14 hospital trusts in England: Overview report*	July 2013	Review	Keogh, B.
*A promise to learn – a commitment to act: Improving the safety of patients in England*	August 2013	Advisory group report	Berwick, D.
*Independent review of deaths of people with a learning disability or mental health problem in contact with Southern Health NHS Foundation Trust from April 2011 to March 2015*	December 2015	Investigation report	Richardson, A. (Mazars)
*Culture change in the NHS: Applying the lessons of the Francis Inquiries*	February 2015	Government response	Department of Health and Social Care
*The report of the Morecambe Bay investigation*	March 2015	Investigation report	Kirkup, B.
*Learning not blaming: Government response to the Freedom to Speak Up Consultation, the Public Administration Select Committee Report Investigating Clinical Incidents in the NHS and the Morecambe Bay Investigation*	July 2015	Government response	Department of Health and Social Care
*Mazars report into mental health and learning disabilities deaths in Southern Health NHS Foundation Trust – a joint response from NHS Improvement, NHS England and the Care Quality Commission*	December 2015	Regulator response	Executive bodies
*Learning, candour and accountability. A review of the way NHS trusts investigate deaths of patients in England*	December 2016	Review	Care Quality Commission
*National guidance on learning from deaths*	March 2017	Guidance	National Quality Board

We purposively sampled policymakers from the Department of Health and Social Care as well as executive bodies including NHS England (NHSE) and Improvement and the CQC to participate in semi-structured interviews. Our approach in formulating a narrative of the evolution of LfD meant that we initially selected individuals who we identified as being directly involved in the conception and development of the policy. Using ‘snowball’ sampling these participants suggested other relevant individuals for interview.^14^ We also identified other stakeholders who had either previously been involved in developing patient safety strategy nationally or were known patient safety ‘experts.’ Interviews were held in the participant’s workplace or over the telephone and lasted between 40–75 minutes. Interviews were conducted between June and September 2019. Informed consent was sought through the provision of a participant information sheet with no refusals to participate.

### Data analysis

Interviews were audio-recorded and transcribed verbatim. Interview data was managed using NVivo version 11.0. We conducted qualitative analysis using a thematic framework approach to code the data and identify patterns and themes.^14^ Initially, open coding was used in the analysis of relevant policy documents. The subsequent coding framework was used alongside the relevant policy and academic literature to inform the development of the interview guide. This coding framework was further adapted inductively with themes arising from the interview data. The researchers independently read and coded initial transcripts and met frequently to discuss emerging themes until agreement on the main themes categorized under the Kingdon framework was reached.

## Results

### Problem stream: Scale of the problem

The publication in 2000 of the UK Department of Health’s (DH) Chief Medical Officer review of patient safety in the NHS, *An organisation with a memory*,^15^ was seminal. With it, came a growing recognition among politicians and the wider public of the scale of health care-related harm.

Highly publicized health care failures in the 1990s and 2000s – including Bristol, Mid Staffordshire, Morecambe Bay and Gosport – had raised concerns that this harm was not being uncovered.^16^ Uncertainty centred not only on the numbers of preventable deaths, but also on the problems in health care that led to these deaths and to the subpopulations most affected. An influential investigation into deaths in people with a learning disability indicated that this population might be particularly vulnerable and that governance mechanisms within mental health and community hospitals were weak in terms of identifying poor quality care.^17^ As one interviewee told us:

Confidential inquiries found that deaths that should’ve been reported, to a coroner, hadn’t been. And deaths that should’ve been taken forward into safeguarding inquiries hadn’t been, to protect people with learning disabilities…One of the recommendations was for a closer look at learning disabilities deaths on a national scale. (Patient safety expert)

As well as concerns about levels of harm within the NHS, investigations of high-profile failures exposed a litany of potentially contributory factors, including poor professional cultures, weak leadership, undue focus on performance targets at the expense of safety issues and failures to listen to concerns of staff and family.^2,3,18^ As a report into the shortcomings at one Trust board noted:

[I]t failed to tackle an insidious negative culture involving a tolerance of poor standards and a disengagement from managerial and leadership responsibilities. This failure was in part the consequence of allowing a focus on reaching national access targets, achieving financial balance and seeking foundation Trust status to be at the cost of delivering acceptable standards of care.^3^^(p3)^

This was accompanied by a lack of transparency, not only in communicating with families but also in approaches to investigations, which lacked depth and independence.^19^ Weak clinical governance systems and leadership insufficiently prioritized patient safety, with safety information – including mortality statistics – being used for assurance rather than scrutinized to pinpoint concerns.^2,3,18,19^ Interviewees remarked on the stream of serious incident reports submitted to the DH where evidence already existed on how these could be prevented, indicating that lessons from patient safety failures were not being learnt.

### Policy stream: Rebalancing learning and measurement

Examination of deaths to drive learning and improvement in the quality of care has a long tradition in the NHS, from local Mortality and Morbidity meetings to investigation of incidents. Confidential Enquiries and the National Patient Safety Agency’s National Incident Reporting and Learning System identify areas for change across the whole NHS. Many other processes from clinical audits to targeted service reviews or inspection by regulators generate information from deaths to support learning and change. These systems of surveillance and review have grown organically, focused on certain specialties or areas of concern and leave unexplored gaps,^9^ which present challenges to developing a systematic understanding of patterns of mortality, preventable harm and quality failings across NHS organizations in order to target improvement efforts.

As far back as the days of Florence Nightingale, deaths have been used as comparative, bench-marking measures for performance monitoring of patient safety. Policymakers have made multiple attempts to use organizational mortality data for this purpose.^10^ The Hospital Standardized Mortality Ratio (HSMR),^11^ became the first widely applied benchmarking measure across acute hospitals during the 2000s. Given the public nature of these data and their use in oversight and regulation, hospital boards became preoccupied with finding ways to improve their ranking, with time devoted to identifying administrative solutions such as changes to coding practices, drawing attention away from identifying and responding to areas of poor care.^19^

By the mid-2000s, academics, clinicians and hospital managers were questioning the utility of measures such as HSMRs to drive improvements, identifying some significant limitations with the approach.^12^ Interviewees revealed that in 2011, when the DH was seeking a new measure to track the progress of hospital safety improvement over time for the NHS National Outcomes Framework, it too held reservations about using measures such as HSMRs or the Summary Hospital-level Mortality Indicator (SHMI). In 2012, a study of 1000 deaths in English NHS acute hospitals (PRISM) based on retrospective case record review (RCRR) identified, for the first time, the scale of preventable deaths in the NHS, estimating around 12,000 such deaths annually.^13^ The DH began to consider if this approach could be used in the National Outcomes Framework.

PRISM gave an estimate to the level of deaths due to problems in care, avoidable mortality, in the NHS…It put a number on how safe the NHS was…an easy to understand number that was assessing the safety of care within acute hospitals. So, we thought, why don’t we explore that?…The creation of a metric and a process to deliver that metric? So, we put a placeholder into the outcomes framework that said we will explore doing just that. (Policymaker)

As the policymakers moved forward with the development of the new indicator, consultation with experts highlighted limitations. Large samples, with multiple reviewers, would be needed to ensure reliability, making this an expensive programme to run.^20^ There were also concerns that this approach would not fully address missed opportunities to learn from deaths in the NHS that were being hindered by fragmented identification and assessment.

A bunch of doctors in a room, having a chat, and the structure to that chat was hugely variable…It was seen as valuable for learning, but there was no structure to that learning. It was reliant on the doctors in that room remembering what their colleague had told them. (Policymaker).

The need to address the lack of a systematized approach to learning from deaths and to rebalance the degree of effort among hospitals dedicated to changing positions in HSMR/SHMI league tables led to a gradual shift in thinking. Interviewees mentioned that a move away from the dominant idea that mortality reviews would be used for safety measurement to one recognizing that the method was more suited to identification of clinical problems, had been suggested by clinicians and academics. With more emphasis on learning, interviewees recalled how they had hoped that an examination of a wider scope of care in hospital settings would result.

Subsequently, two new national programmes focussed on learning were commissioned, the Learning Disabilities Mortality Review Programme (LeDeR) (2015) and the National Mortality Case Record Review (NMCRR) programme (2016). The NMCRR introduced a standardized approach to case identification and review of care provided before death in acute hospitals.^6^ LeDeR took a broader view, examining a longer period before death, with input from multiple agencies and family members.^21^

### Political stream: The impact of policy entrepreneurs

Kingdon describes a policy entrepreneur as someone who uses his or her knowledge of processes to promote their own policy objectives.^8^ Several interviewees highlighted the role of Jeremy Hunt (Secretary of State for Health and Social Care, 2012–2018) as a major proponent in the development of the national patient safety agenda, acting as a policy entrepreneur in the development of LfD. His time in office coincided with the publications of inquiries and investigations into Mid Staffordshire, Morecambe Bay and Southern Health.^3,18,22^ Interviewees explained how Hunt’s desire to develop better methods for understanding safety in the NHS, prompted an interest in the approaches used in industries such as aviation, as well as in traditional health care settings. Hunt visited the Virginia Mason Institute in the United States, an organization known for its culture of learning, quality improvement and openness. This was pivotal in heightening his interest in the transparent use of performance indicators to drive safety improvement.

Mr Hunt was a firm believer in the power of transparency and exposing variation through data…Things like league tables, indicators and producing top tens, demonstrate to me that he fundamentally believed that if you show everybody the range of variation and performance of whatever exists, and expose it, and make it really uncomfortable to be at the bottom, and then you drive the [whole]. (Policymaker)

Interviewees remarked that when an enlarged PRISM study^20^ (total 34 English acute Trusts) showed a lack of association between HSMR/SHMI measures and avoidable health care-related deaths identified by mortality review, Hunt acknowledged the limitations of these measures. But he continued to believe that publicly available comparative data on avoidable deaths for each NHS hospital would be a powerful stimulus for improvement. Interviewees were keen to emphasise how he maintained this position despite concerns from leading clinicians and academics that such data are likely to be misleading both due to measurement biases and the accuracy of comparisons, given the small numbers of such deaths normally found.

Several families affected by patient safety failures worked assiduously to better understand the circumstances that led to serious harm and death of their loved ones. Among them were James Titcombe, who successfully lobbied for an independent inquiry at the Morecambe Bay Trust, and Sara Ryan, who called for an independent investigation at Southern Health.^23,24^ Taken together, families affected by patient safety failures can be seen as another policy entrepreneur. Interviewees mentioned how these families contributed to bringing NHS safety concerns to the notice of politicians while also seeking reassurance that measures were in place to avoid the recurrence of such problems. Affected families also pressured DH and NHS leaders to hold organizations to account for failures.

Interviewees explained how Jeremy Hunt expressed deep concern for the affected families and requested greater responsiveness from the NHS in providing explanations of what went wrong. However, many remained sceptical that this would happen. As one interviewee told us:

The guidance will land in a Trust but will only be successful if you’ve got the right culture and leadership to embrace the spirit and the [fidelity] of that guidance…Families have repeatedly told us…when they challenged the factual accuracies of the independent investigation, they were seen as vexatious complainants. Really, really difficult. Couldn’t get past the Trust board, couldn’t get past the governance or compliance team. (Patient safety expert)

### Confluence of the issues listed above

Interviewees suggested that a policy window appeared in the aftermath of the publication of the independent investigation into Southern Health,^22^ which exposed the Trust’s failure to provide a systematic approach to reviewing deaths where there was potential for significant learning. The investigation also identified many of the issues that had arisen in previous health care scandals, including poor leadership and failures of accountability which, in this Trust, had resulted in only 1% of unexpected deaths among patients with learning disabilities undergoing investigation.

In an effort to determine whether these issues were ubiquitous to the NHS, Jeremy Hunt commissioned the CQC to undertake a review across a range of NHS organizations. The subsequent report *Learning, candour and accountability*, published in 2016, confirmed the fragmented and at times limited approach to reviewing and investigating deaths, especially in mental health and learning disabilities.^5^ The report made wide-ranging recommendations for improvement, including the implementation of a single framework for learning from deaths across all NHS organizations, incorporating guidance on governance, sharing learning and involvement of families. These were accepted in full by the DH:

The key contributory factor was the CQC report that was commissioned off the back of the problems at Southern Health, which showed that the NHS did not have a clear policy for how it learned from the deaths…Mr Hunt stood up in the House on the day it was published and said that the NHS would have a consistent policy to learn from deaths by April 2017 … which then precipitated some of the fastest policymaking I’ve seen. (Policymaker).

Interviewees outlined the various discussions held at this juncture between key stakeholders in the DH and the other executive bodies. Following the CQC report, the DH asked the NQB, then under the joint chairmanship of the Medical Director of NHSE and the chair of CQC, to develop a policy that addressed each of the recommendations. Jeremy Hunt continued to press for publication of the numbers of deaths attributable to problems in care. This position created tensions among those charged with policy creation. At the time, the NQB commented on the need to balance any mortality data component of the policy with the focus on learning. Interviewees described how the policy – built on learning-focused initiatives already in place such as NMCRR and LeDeR - was obliged to incorporate the additional requirement for publication of mortality data in Quality Accounts (report about the quality of services offered by an NHS organization), despite views among some individuals in the executive agencies at the time that this could be counterproductive.

Addressing wider governance issues, a Non-Executive Director in each NHS organization was to be assigned the role of scrutinizing implementation and increasing the profile of the programme at Board level.^22^ The emphasis on engagement and disclosure with families and carers was seen as an imperative. Interviewees referred to the direct involvement of families throughout the process of policy development as a mechanism for ensuring their concerns were kept under consideration.

Following a statement by Jeremy Hunt to Parliament in late 2016 outlining the plans for a mortality focussed programme for NHS organizations, in March 2017 the NQB published the national LfD Framework.^4^

## Discussion

Applying Kingdon’s MSA has enabled us to obtain a clearer understanding of the narrative for the evolution of the LfD policy. Two interacting factors came together to drive the issue of health care-related death up the political agenda. The first was a long-standing concern that poor care and patient harm were being missed. This factor was heightened following a succession of high-profile failures, with the concomitant loss of opportunities to improve quality of care. The revealing of the poor investigation into deaths at Southern Health, coupled with the acknowledgement that the lack of systematic examination of deaths was widespread across the NHS, opened a window for policy action.^5,22^

The LfD policymaking process shares common features with previous national patient safety policies which impact on performance. Powell’s analysis of DoC,^25^ based on MSA, also highlights how a long-term concern (the need for transparency with patients and families after harm) only reached the policymaking agenda in the aftermath of an NHS failure (Mid Staffordshire).^3^ Competing goals within a policy, emerging from attempts multiple issues, can undermine each other. For LfD there is a clear tension within the policy between the desire to develop a culture of reflection and learning around deaths and the need for accountability and performance management (the requirement to publish rates of deaths due to problems in care). For DoC, the broad expectation that the policy could change both culture and behaviour was limited by policy framing and regulatory oversight. This resulted in the process becoming a ‘tick box’ exercise within some NHS organizations, while obfuscating the balance between individual professional and organizational responsibilities.^26^

In a similar way, the National Incident Reporting and Learning System was established following revelations of the scale of harm in the NHS, and was designed to promote learning from that harm.^15^ Inherent tensions between perceptions of incident occurrence as patient safety failures and high organizational reporting rates seen as an indicator of good safety culture, have resulted in a system generating over a million incident reports annually, the majority related to ‘no harm’ events and few reporting common adverse events.^27^ Failure to utilize the signals from ‘no harm’ near misses or to adequately capture the scale of harmful events are generally acknowledged to arise from a lack of capacity for openness in inquiry, investigation and learning.^28^

This leads us to the second important factor influencing design and implementation: the role of policy entrepreneurs – in this case, Jeremy Hunt and families affected by safety failures. Policymakers had become convinced of the limits of mortality measures to track safety at both local and national levels. However, Jeremy Hunt remained a keen advocate of their use. Guldbrandsson K et al. ^29^ describe several attributes of a policy entrepreneur which were exemplified by Jeremy Hunt and the affected families. Jeremy Hunt’s credibility and authority ensured he had a ‘claim to hearing’ and the ability to leverage his ‘political connections.’ The ‘sheer persistence’ of families in voicing the appalling experiences of patients at the hands of the NHS was also significant. Affected families pursued more rigour in the investigation and transparency in reporting of deaths in NHS Trusts, with Hunt as the focus for their advocacy. While agreeing to support affected families, Hunt also promoted the use of RCRR for both learning about care quality and enhancing performance management. In the creation of this policy, the policy entrepreneurs were the prominent factor in the introduction of policy elements that may be at odds with the current consensus, such as the value of publication of deaths caused by problems in care.

Our analysis demonstrates the complexity of policymaking in patient safety, often driven by expedient responses to prominent health care failings. Whilst the Southern Health review findings principally concerned the quality and reporting standards for investigations of unexpected deaths among people with a learning disability, the LfD policy that emerged promoted a broader surveillance of clinical care for improvement and the publication of data on organizational avoidable deaths to encourage transparency. Furthermore, expediency encouraged policymakers to draw into the policy existing programmes that aligned with its aims (in this case, LEDER and NMCRR), despite neither being formally evaluated.

## Limitations

There are two main limitations with this study. The first is the several critiques of the MSA approach that exist.^30^ These critiques include the tendency for MSA to conflate agenda setting and decision-making processes, the lack of standardization in interpretation of components and subcomponents and the vague definition of a policy entrepreneur. Such issues, taken together, may have hindered our analysis in unpacking the complexity of factors that contributed to the development of LfD. Nevertheless, we captured a range of perspectives from both key informants involved with the development of the policy and long-standing experts in the field. This provided a nuanced understanding of policymaking that would not have been obtained from the analysis of policy documents alone.

The second limitation is the relatively small numbers of interviewees, the majority of whom were policymakers providing elite accounts.^14^ We acknowledge that, had we interviewed a larger number of individuals, some of those individuals may have expressed alternative views to those reported in this paper.

## Conclusions

The problems that patient safety policies seek to address may have been recognized for many years, only reach the top of the policy agenda at a time of crisis. The policies that emerge tend to be multi-faceted, responding to a range of issues, not only those arising from the investigations of failures but also to others causing concern across the system at the time. Using ‘off-the-shelf ‘solutions (NMCRR, LeDeR, DoC) to generate the new policy and accelerate its delivery may have some advantages – rationalizing discrete programmes with which organizations are already familiar under a single umbrella programme while mitigating some of the negative impacts of ‘top-down’ policymaking, increasing legitimacy, feasibility and support.^31^ However, with few of these policy components ever having been formally evaluated, their likely outcomes or those of the policy as a whole become difficult to predict, particularly as they become layered on top of others.

A future where patient safety policymaking is based on evidence of successful interventions is desirable but, given the politically sensitive nature of safety issues in the NHS, this is unlikely to happen. In these circumstances, it would seem imperative to continue to examine the policymaking process in depth, in order to predict potential weaknesses and identify aspects that could be strengthened to increase the likelihood that goals will be met. Equal attention to creating a receptive milieu within the NHS through continued focus on the promotion of organizational learning from patient safety, supported by senior leaders who take ownership for developing a learning culture locally, is also necessary.
